# Emergent bedside management of a pneumatocele in a neonate via Fogarty catheter

**DOI:** 10.1002/ccr3.8331

**Published:** 2023-12-13

**Authors:** Michael C. Larkins, John Iasiello, Sunny R. Cai, Michael Holdeman, John Narron, Adrienne L. Hutton

**Affiliations:** ^1^ East Carolina University Brody School of Medicine Greenville North Carolina USA; ^2^ East Carolina Anesthesia Associates Greenville North Carolina USA

**Keywords:** Fogarty catheter, neonatal ventilation, pneumatocele

## Abstract

Persistent pneumatoceles in neonates increase mortality, and little literature regarding emergent treatment in a decompensating patient exists. We present the emergent management of a pneumatocele in a decompensating neonate by isolation with a Fogarty catheter.

## Graphical abstract

Chest radiograph showing contrast in Fogarty catheter to visualize placement for isolation of pneumatocele in the right lung of a neonate.

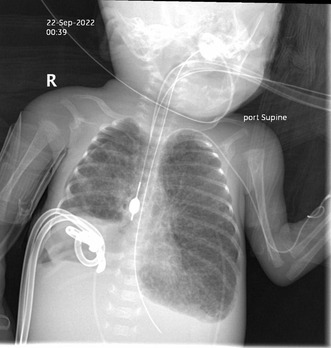

## 1 INTRODUCTION

Pneumatoceles result from air leaking into damaged lung parenchyma and are a known complication of positive‐pressure mechanical ventilation in neonates. Pneumatoceles have been found to be congenital, traumatic, ventilator‐induced, and have also been found after hydrocarbon ingestion; the most common cause of pneumatocele in neonates is the congenital variety.^1^ The use of surfactant and modern ventilation techniques have led to a decreased incidence. While most resolve spontaneously, persistent pneumatoceles are associated with higher mortality.^2^ Additionally, increased incidence of secondary outcomes such as pneumonia has been reported. Therapeutic interventions, such as lateral decubitus positioning, unilateral lung intubation, and ultrasound‐guided percutaneous drainage, have been reported.^3^ Contemporary management of pneumatoceles involves diagnosis with radiography followed by an initial attempt of surfactant and gentle ventilation, though this may not be sufficient in more advanced pneumatoceles.^2^ Management of neonatal pneumatoceles via a Fogarty catheter has been reported.^4^ However, there is sparse literature regarding the emergent treatment of pneumatoceles in rapidly decompensating neonates. We present a novel management of a complicated pneumatocele by emergent insertion of a Fogarty catheter.

## 2 CASE PRESENTATION

**FIGURE 1:**
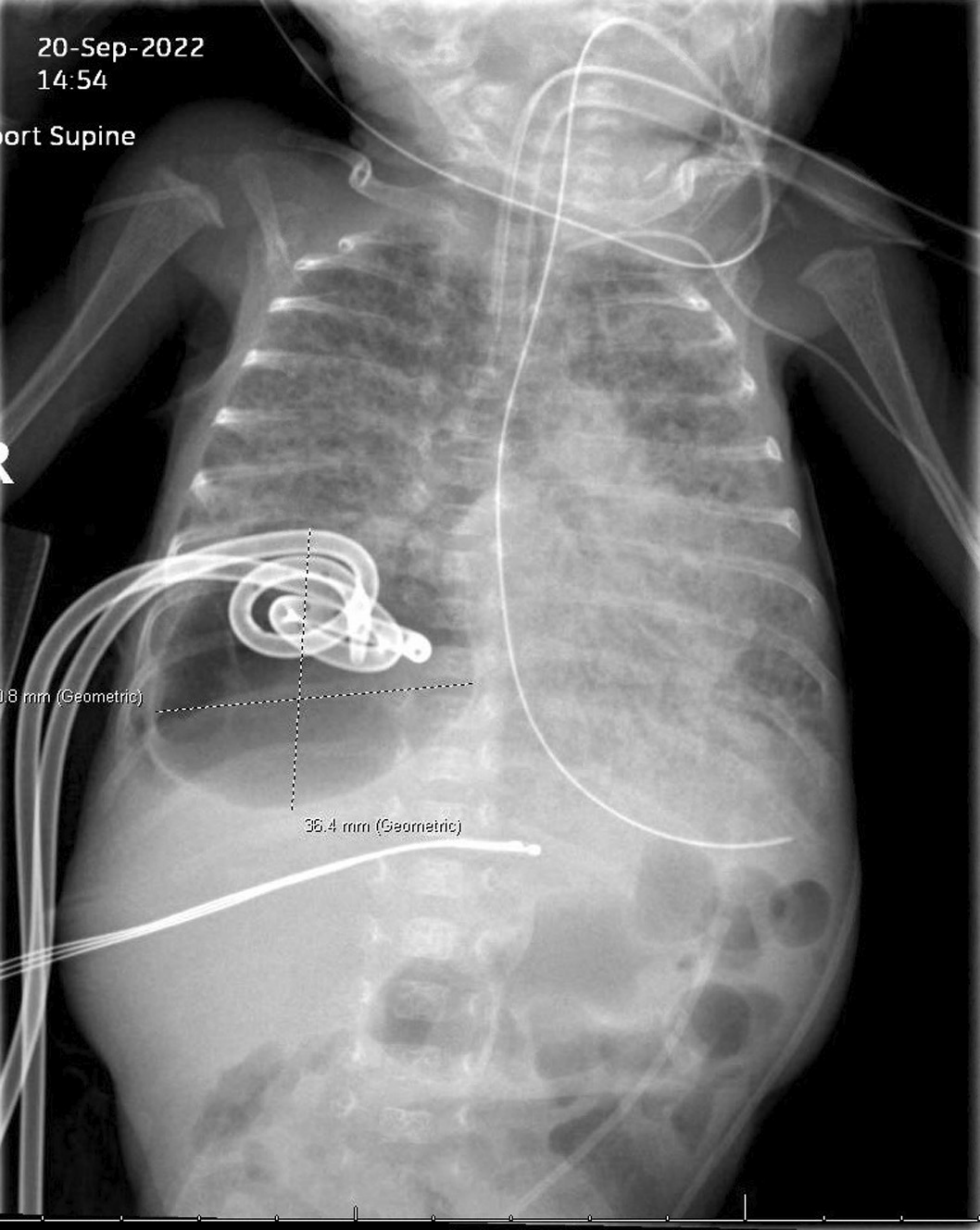
Initial bedside imaging showing pneumatocele with two thoracostomy tubes in place.

The pediatric anesthesiology service at a rural tertiary care center was emergently consulted for the management of a 5‐week‐old, 1‐kg hypoxic male with known persistent pneumatocele in the right lung base. The patient was born at 29 weeks and 2 days via cesarean section; antenatal steroids were reportedly given. The patient had a history of hyperbilirubinemia and was undergoing phototherapy for a total bilirubin peak of 6.0 mg/dL. On initial assessment, the neonate had two 8.5 F pigtail thoracostomy tubes in place for management of hypoxia—one in the anterior pleural space with a small one‐chamber leak and one in the posterior pleural space with a continuous five‐chamber leak (see Figure 1). The patient was intubated with a 3.0 uncuffed endotracheal tube (ETT). The patient was experiencing desaturation events to 50%–60% with associated hypotension due to high‐volume air loss from the posterior chest tube. Clamping of the tube resulted in short‐term improvement in the patient's oxygen saturation; within minutes of clamping the patient's saturation would begin to again dip into the 50%–60% range. This cycle was ongoing as the anesthesia team assessed the patient.

**FIGURE 2:**
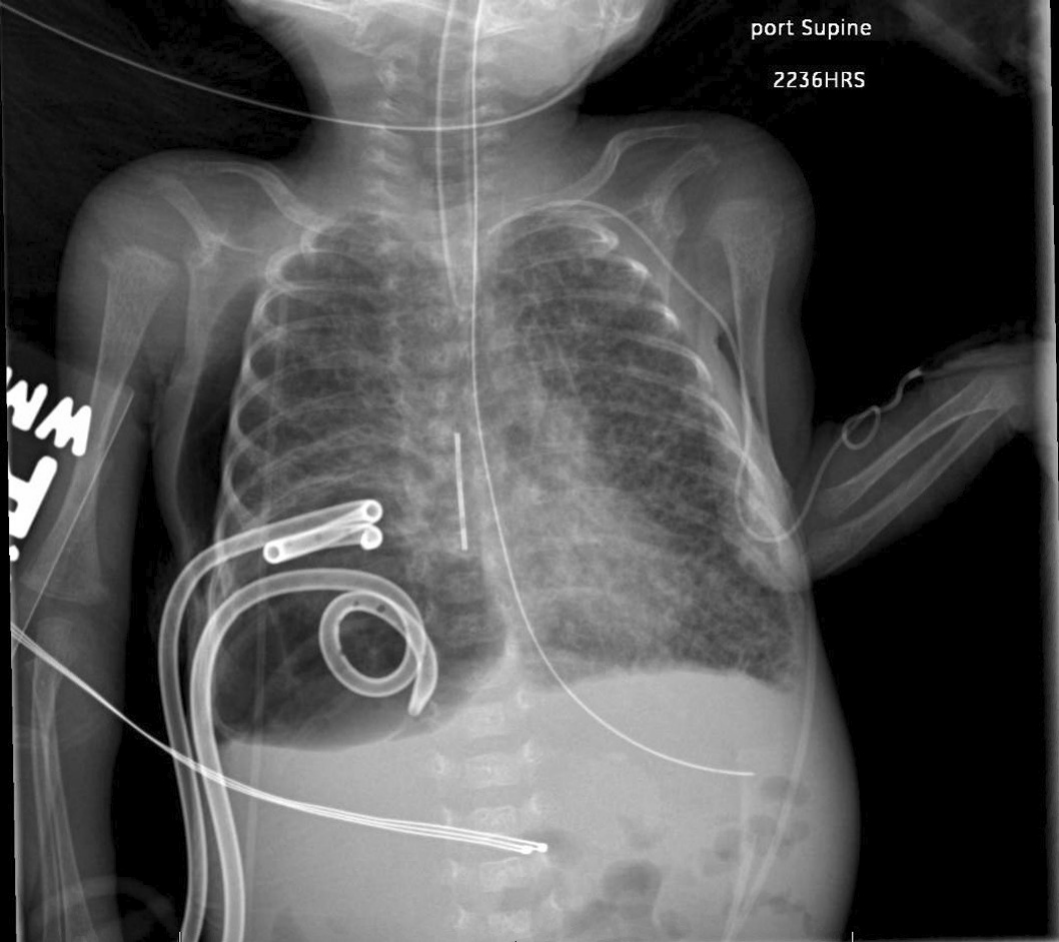
Follow‐up imaging at bedside after placement of Fogarty catheter within pneumatocele with subsequent pneumatocele decompression.

Upon initial evaluation, it was determined the ETT was too large to advance and mainstem for one‐lung ventilation. The anesthesia team emergently decided to utilize a Syntel® silicone embolectomy catheter with a 3 Fr Fogarty balloon to selectively isolate the distal portion of the patient's lung affected by the pneumatocele. After time‐out was called, the 3 Fr catheter was passed into the patient's trachea next to the in‐place ETT. Due to the limited space available between the patient's vocal cords, the inter‐cord space between the ETT and tubercle of the epiglottitis was utilized. The catheter was advanced until resistance was felt. Catheter placement in the pneumatocele was confirmed via chest radiograph (see Figure 2).

The catheter was then pulled back slightly, and the patient's head was turned to the side of the unaffected lung. The catheter was then advanced further, past the ETT, in the hope it would preferentially occlude the desired primary bronchus, proximal to the portion of the patient's lung affected by the pneumatocele; success was ultimately achieved, and placement was confirmed via radiography. After placement was confirmed, the balloon was inflated with 1 mL of air, and the catheter was placed under tension with gentle superior force. While the leak from the posterior chest tube was still present, the air leak decreased from five to two chambers. The patient's oxygen saturation improved to 98. The anterior chest tube was left on −20 mmHg, and the posterior chest tube was put to water seal. In conjunction with the neonatal intensive care unit team, it was decided to leave the catheter in place for the time being.

**FIGURE 3:**
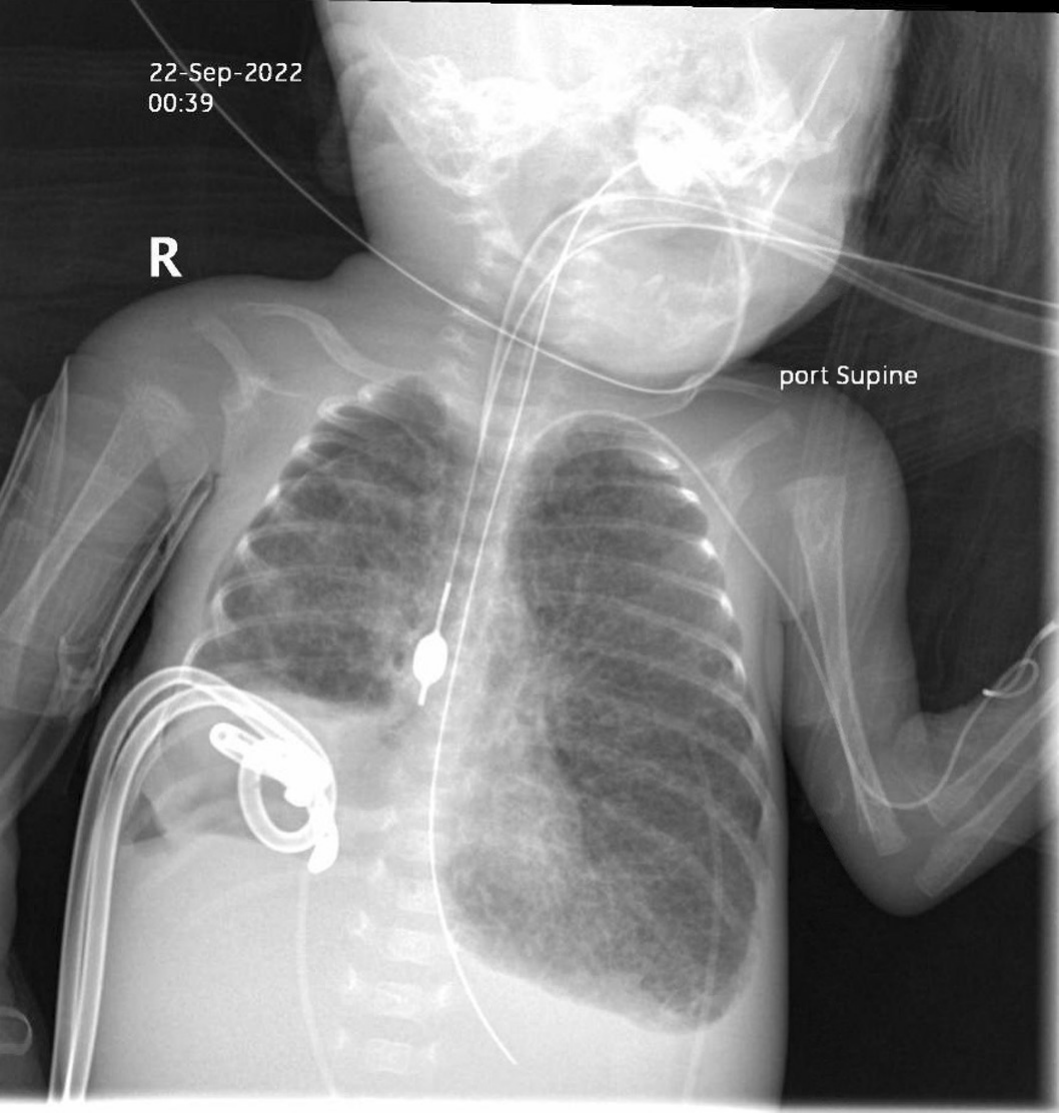
Contrast in Fogarty catheter to visualize placement for isolation of pneumatoceles in right lung.

Two days after the Fogarty catheter was placed; the patient began experiencing repeated desaturation events. The catheter's position was seen still in place under fluoroscopy. The cause of the repeated desaturations was assumed to be the result of a secondary and more proximal pneumatocele. It was decided that the best way to temporize the situation was to pull the catheter back into the right bronchus, proximal to the affected lung areas. This was accomplished by putting radiology contrast dye into the catheter balloon and taking a portable chest radiograph. The measurement feature was used on the machine to determine the distance to withdraw the catheter; the balloon was deflated, and the catheter was moved superiorly and placed proximal to the patient's pneumatocele, with immediate improvement in oxygenation. The balloon was subsequently reinflated slowly until bubbling occurred in the patient's chest tubes, and a minimal amount of contrast was reinjected to stop the bubbling and ensure a seal had been obtained; positioning was confirmed via repeat chest radiograph (see Figure 3). Unfortunately, the patient continued to require increasing vasopressor support and had worsening acidosis; resulting blood gas showed a pH of 7.22 and a bicarbonate value of 17 mmol/L. The patient ultimately expired due to cardiorespiratory arrest due to pulmonary hypertension and metabolic acidosis; repeat arterial blood gas measurements continued to show both down‐trending pH and bicarbonate values. Informed consent was obtained from the patient's mother for publication of this case report.

## 3 DISCUSSION

The incidence of pneumatoceles has decreased dramatically since the advent of surfactant and with continued advances in neonatal ventilation. Interventional decompression of pneumatoceles is generally not necessary unless a patient is unstable, as was the case in our patient. While surgical options were available, this patient was unlikely to tolerate lower lobectomy or functional pneumonectomy. Therefore, their decompensating clinical condition warranted a rapid nonsurgical approach. Unilateral intubation was perceived as inadequate to successfully ventilate the patient, and there were concerns for the in‐situ endotracheal tube being too large for advancing to mainstem. Ventilation with high‐frequency oscillation was also considered inadequate but not attempted. Management with two chest tubes was unsuccessful due to rapid re‐accumulation of air within the patient's pneumatocele and thorax; these tubes were noted to have managed the initial mass effect from the pneumatocele though. This failure was considered to be the result of air flowing from the ETT and preferentially though the patient's affected lung, then proceeding through the pneumatocele and directly out of the chest cavity via the chest tubes, reducing the ventilation of the contralateral lung. This explains the back‐and‐forth seen initially in our patient, in which clamping the patient's chest tube would temporarily improve oxygen saturations until enough air reaccumulated in the lung affected by the pneumatocele to compress the mediastinum and contralateral lung.

Insertion of a Fogarty catheter has been described in the operating room setting, but emergent bedside insertion into the pneumatocele alongside an in‐situ ETT is a technique not previously reported in the literature. A case series by Malik et al. does report and illustrate the use of a Fogarty catheter to successfully decompress a pneumatocele in multiple neonates.^4^ However, both patients in this case series were not managed at bedside, nor were their catheters placed alongside a preexisting ETT. There are novel benefits to our technique. First, by passing the catheter next to the ETT, the patient remained intubated which improved the safety profile of the procedure and does not expose the patient to the unnecessary risk of extubation and reintubation. Second, deflation of the balloon allowed for reassessment and adjustment of the catheter position, arguably more safely than attempting to isolate an entire lung; during whole lung isolation, it is possible for the balloon to be moved too far superiorly and ultimately block the patient's trachea. Third, the addition of contrast dye to the catheter balloon assisted with imaging and confirmed placement. The balloon was then reinflated to selectively block portions of the lung and isolate the pneumatocele. This combination of plain radiography and the measurement feature of portable radiography to the authors knowledge has also never been reported in the literature.

While our patient ultimately expired due to exhaustion and difficulty securing the tube, catheter insertion produced significant improvements in the patient's oxygen status. Our patient was successfully temporized, and it is possible had the pediatric anesthesia team been involved earlier in our patient's course, earlier catheter insertion may have stabilized the patient beyond a few days and perhaps may even have been a bridge to surgical intervention, such as the aforementioned lobectomy or pneumonectomy.

Additionally, it should be mentioned that our patient was not considered a candidate per the pediatric surgery service and our institutional policy. Current literature reports a relative contraindication to ECMO for patients less than 34 weeks gestational age or less than two kg, as was the case with our patient.^5^

Key strengths of our study include its novelty and utility for patients not in an operating room or intensive care unit. The environment in which it can be utilized makes studying the application of this technique to more patients potentially more streamlined, though we can only advocate for this technique to be applied to patients without other, safer options. Key limitations of this case report include the limited sample size and lack of control for factors such as patient stability. The assumption with our intervention is that there is potential for it to be used as a last‐ditch effort to potential save or at least temporize patients in similar situations, though the severity of our patient on initial presentation to our service and ultimate death muddle interpretation of this assumption.

In conclusion, pneumatoceles are a known source of increased neonatal mortality. Their successful management via modified patient position, unilateral lung intubation, percutaneous catheter placement, and surgical management. While management of pneumatocele via Fogarty catheter placement has been reported in the literature, the technique described in this case report uniquely describes a successful bedside placement and with an existing endotracheal tube in place to continuously maintain an airway.

## AUTHOR CONTRIBUTIONS

**Michael C. Larkins:** Data curation; formal analysis; investigation; project administration; resources; validation; visualization; writing – original draft; writing – review and editing. **John Iasiello:** Formal analysis; investigation; project administration; validation; visualization; writing – original draft; writing – review and editing. **Sunny R. Cai:** Conceptualization; investigation; methodology; supervision; validation; writing – original draft; writing – review and editing. **Michael Holdeman:** Conceptualization; formal analysis; methodology; project administration; validation; visualization; writing – original draft; writing – review and editing. **John Narron:** Conceptualization; investigation; project administration; supervision; validation; writing – original draft; writing – review and editing. **Adrienne L. Hutton:** Conceptualization; formal analysis; investigation; methodology; project administration; supervision; validation; visualization; writing – original draft; writing – review and editing.

## FUNDING INFORMATION

No external funding declared.

## CONFLICT OF INTEREST STATEMENT

No competing interests declared.

## CONSENT

Written informed consent was obtained from the patient's parent to publish this report in accordance with the journal's patient consent policy.

## Data Availability

The data that support this case report is available from corresponding author upon request.

## References

[1] Hussain N , Noce T , Sharma P , et al. Pneumatoceles in preterm infants—incidence and outcome in the post‐surfactant era. J Perinatol. 2010;30(5):330‐336. 19812584 10.1038/jp.2009.162

[2] Arora P , Kalra VK , Natarajan G . Pneumatoceles in infants in the neonatal intensive care unit: clinical characteristics and outcomes. Am J Perinatol. 2013;30(8):689‐694. 23283803 10.1055/s-0032-1331028

[3] Hameedi S , Hu H , Angueyra C , Kabagambe S , Duron V , Parravicini E . Management of pneumatocele in an extremely low birth weight neonate with percutaneous pigtail catheter by ultrasound guidance. J Pediatr Surg Case Rep. 2022;76:102075.

[4] Malik I , Puvabanditsin M , Memon N , Khan I , Stahl GE . Use of a Fogarty balloon catheter to decompress pneumatocele in preterm neonates. OA Case Reports. 2013;2(15):148.

[5] Roeleveld PP , Mendonca M . Neonatal cardiac ECMO in 2019 and beyond. Ann Front Pediatr. 2019;7:327. 10.3389/fped.2019.00327 PMC6712998 31497583

